# Tetra-μ-acetato-κ^4^ *O*:*O*′;κ^3^ *O*,*O*′:*O*′;κ^3^ *O*:*O*,*O*′-bis­[(acetato-κ^2^ *O*,*O*′)(1,10-phenanthroline-κ^2^ *N*,*N*′)europium(III)]

**DOI:** 10.1107/S1600536810015680

**Published:** 2010-05-08

**Authors:** Wen-Jing Liu, Zhao-Yang Li, Zhi-Qiang Wei, Shan-Tang Yue

**Affiliations:** a School of Chemistry and Environment, South China Normal University, Guangzhou 510006, People’s Republic of China

## Abstract

In the title centrosymmetric dinuclear Eu^III^ complex, [Eu_2_(CH_3_COO)_6_(C_12_H_8_N_2_)_2_], each Eu^III^ cation is coordinated by seven O atoms from five acetate anions and two N atoms from one phenanthroline ligand in a distorted tricapped trigonal-prismatic geometry. Four acetate anions bridge two Eu^III^ cations to form the dinuclear complex, with an Eu⋯Eu distance of 3.9409 (8) Å. Weak inter­molecular C—H⋯O hydrogen bonding is present in the crystal structure.

## Related literature

For related lanthanide complexes with 1,10-phenanthroline and acetate ligands, see: Hu *et al.* (2006 ▶); Panagiotopoulos *et al.* (1995 ▶).
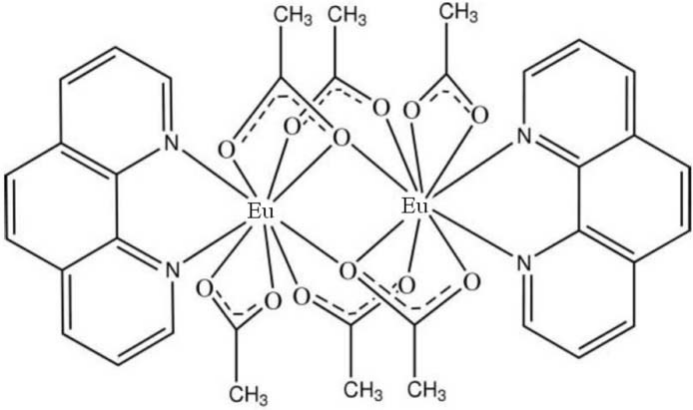


## Experimental

### 

#### Crystal data

[Eu_2_(C_2_H_3_O_2_)_6_(C_12_H_8_N_2_)_2_]
*M* *_r_* = 1018.61
Triclinic, 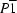

*a* = 8.7671 (19) Å
*b* = 8.9265 (19) Å
*c* = 12.992 (3) Å
α = 103.631 (2)°
β = 109.254 (2)°
γ = 98.300 (3)°
*V* = 905.1 (3) Å^3^
*Z* = 1
Mo *K*α radiation
μ = 3.50 mm^−1^
*T* = 298 K
0.20 × 0.19 × 0.18 mm

#### Data collection

Bruker SMART CCD diffractometer
Absorption correction: multi-scan (*SADABS*; Bruker, 2001 ▶) *T*_min_ = 0.541, *T*_max_ = 0.571
5010 measured reflections
3474 independent reflections
3062 reflections with *I* > 2σ(*I*)
*R*_int_ = 0.021

#### Refinement

*R*[*F* ^2^ > 2σ(*F* ^2^)] = 0.030
*wR*(*F* ^2^) = 0.062
*S* = 1.05
3474 reflections
247 parameters
H-atom parameters constrained
Δρ_max_ = 0.80 e Å^−3^
Δρ_min_ = −0.64 e Å^−3^

### 

Data collection: *SMART* (Bruker, 2001 ▶); cell refinement: *SAINT* (Bruker, 2001 ▶); data reduction: *SAINT*; program(s) used to solve structure: *SHELXTL* (Sheldrick, 2008 ▶); program(s) used to refine structure: *SHELXTL*; molecular graphics: *SHELXTL*; software used to prepare material for publication: *SHELXTL*.

## Supplementary Material

Crystal structure: contains datablocks I, global. DOI: 10.1107/S1600536810015680/xu2753sup1.cif

Structure factors: contains datablocks I. DOI: 10.1107/S1600536810015680/xu2753Isup2.hkl

Additional supplementary materials:  crystallographic information; 3D view; checkCIF report

## Figures and Tables

**Table 1:** Hydrogen-bond geometry (Å, °)

*D*—H⋯*A*	*D*—H	H⋯*A*	*D*⋯*A*	*D*—H⋯*A*
C2—H2⋯O2^i^	0.93	2.57	3.287 (6)	135
C12—H8⋯O6^ii^	0.93	2.44	3.078 (6)	126
C16—H10*C*⋯O1^iii^	0.96	2.45	3.390 (6)	165

Symmetry codes: (i) 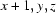; (ii) 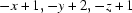; (iii) 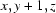.

**Fig. 1.**
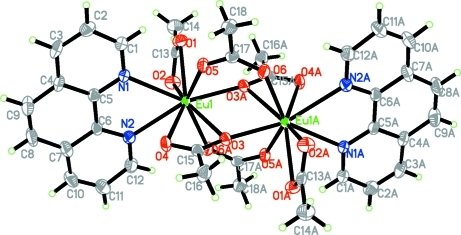
Displacement ellipsoid plot (40% probability level) of the title compound [symmetry code: (A) -x+1, -y+2, -z+1].

## supplementary crystallographic information

### Comment

Dinuclear lanthanide complexes with 1,10-phenanthroline and acetate ligands had previously been reported (Panagiotopoulos *et al.*, 1995; Hu *et al.*, 2006). In this title complex, each Eu atom is coordinated by two N atoms from one chelating phenanthroline ligand and seven oxygen atoms from acetate ions, to form a distorted tricapped trigonal prism, giving a dimeric structure with an inversion center (Fig.1). The result of the dinuclear centrosymmetric molecule with the Eu···Eu distance of 3.9409 (8) Å was that acetate ions exhibit three different coordination modes: common bidentate chelating mode, bidentate bridging mode and tridentate bridging mode. The Eu1—O bond distances vary from 2.359 (3) Å to 2.586 (3) Å and the Eu1—N bond length are 2.594 (3) Å and 2.649 (4) Å. The C—O distances of CH_3_COO^-^ are within the range of 1.257 (5)Å to 1.273 (5) Å. This complex exhibits a three-dimensional structure via C—H···O hydrogen-bonds (Table 1).

### Experimental

A stoichiometric amount of acetic acid and a quantitative amount of 1,10-phenanthroline (0.5 mmol) were mixed and then dissolved in 95% ethanol solution (20 ml). The pH value of the solution was adjusted to 6.5 by adding 1.0 M NaOH solution, and then added dropwise to the ethanol solution (20 ml) of Eu(NO_3_)_3_.6H_2_O (0.5 mmol). The solution mixture was stirred continuously for 2 h at room temperature and then filtered. Single crystals were obtained by evaporation after one week.

### Refinement

H atoms were positioned in calculated positions, with C—H = 0.93 (aromatic) and 0.96 Å (methyl), and refined in riding mode with U_iso_(H) = 1.5U_eq_(C) for methyl and 1.2U_eq_(C) for the others.

### Crystal data

**Table:**

[Eu_2_(C_2_H_3_O_2_)_6_(C_12_H_8_N_2_)_2_]	*Z* = 1
*M**_r_* = 1018.61	*F*(000) = 500
Triclinic, *P*1	*D*_x_ = 1.869 Mg m^−^^3^
Hall symbol: -P 1	Mo *K*α radiation, λ = 0.71073 Å
*a* = 8.7671 (19) Å	Cell parameters from 2079 reflections
*b* = 8.9265 (19) Å	θ = 0.7–25.2°
*c* = 12.992 (3) Å	µ = 3.50 mm^−^^1^
α = 103.631 (2)°	*T* = 298 K
β = 109.254 (2)°	Block, colorless
γ = 98.300 (3)°	0.20 × 0.19 × 0.18 mm
*V* = 905.1 (3) Å^3^	

### Data collection

**Table:**

Bruker SMART CCD diffractometer	3474 independent reflections
Radiation source: fine-focus sealed tube	3062 reflections with *I* > 2σ(*I*)
graphite	*R*_int_ = 0.021
ω scans	θ_max_ = 26.0°, θ_min_ = 1.7°
Absorption correction: multi-scan (*SADABS*; Bruker, 2001)	*h* = −10→10
*T*_min_ = 0.541, *T*_max_ = 0.571	*k* = −8→10
5010 measured reflections	*l* = −14→15

### Refinement

**Table:**

Refinement on *F*^2^	Primary atom site location: structure-invariant direct methods
Least-squares matrix: full	Secondary atom site location: difference Fourier map
*R*[*F*^2^ > 2σ(*F*^2^)] = 0.030	Hydrogen site location: inferred from neighbouring sites
*wR*(*F*^2^) = 0.062	H-atom parameters constrained
*S* = 1.05	*w* = 1/[σ^2^(*F*_o_^2^) + (0.025*P*)^2^] where *P* = (*F*_o_^2^ + 2*F*_c_^2^)/3
3474 reflections	(Δ/σ)_max_ < 0.001
247 parameters	Δρ_max_ = 0.80 e Å^−^^3^
0 restraints	Δρ_min_ = −0.64 e Å^−^^3^

### Special details

**Table:**

Geometry. All esds (except the esd in the dihedral angle between two l.s. planes) are estimated using the full covariance matrix. The cell esds are taken into account individually in the estimation of esds in distances, angles and torsion angles; correlations between esds in cell parameters are only used when they are defined by crystal symmetry. An approximate (isotropic) treatment of cell esds is used for estimating esds involving l.s. planes.
Refinement. Refinement of *F*^2^ against ALL reflections. The weighted *R*-factor wR and goodness of fit *S* are based on *F*^2^, conventional *R*-factors *R* are based on *F*, with *F* set to zero for negative *F*^2^. The threshold expression of *F*^2^ > σ(*F*^2^) is used only for calculating *R*-factors(gt) etc. and is not relevant to the choice of reflections for refinement. *R*-factors based on *F*^2^ are statistically about twice as large as those based on *F*, and *R*- factors based on ALL data will be even larger.

### Fractional atomic coordinates and isotropic or equivalent isotropic displacement parameters (Å^2^)

**Table:**

	*x*	*y*	*z*	*U*_iso_*/*U*_eq_	
Eu1	0.47430 (3)	0.84412 (3)	0.356229 (17)	0.02341 (8)	
O3	0.5788 (4)	1.1409 (4)	0.4756 (2)	0.0312 (7)	
O4	0.6208 (4)	1.0735 (4)	0.3157 (2)	0.0336 (7)	
O2	0.2565 (4)	0.5955 (4)	0.2687 (3)	0.0419 (8)	
O1	0.5000 (4)	0.5829 (4)	0.3834 (3)	0.0373 (8)	
O5	0.7449 (4)	0.8969 (4)	0.4988 (2)	0.0310 (7)	
N1	0.6375 (4)	0.7194 (4)	0.2384 (3)	0.0277 (8)	
C1	0.7671 (6)	0.6658 (5)	0.2863 (4)	0.0362 (11)	
H1	0.7915	0.6637	0.3612	0.043*	
C2	0.8702 (6)	0.6116 (6)	0.2307 (4)	0.0423 (12)	
H2	0.9609	0.5753	0.2678	0.051*	
C3	0.8341 (6)	0.6134 (6)	0.1215 (4)	0.0450 (13)	
H3	0.9007	0.5782	0.0829	0.054*	
C4	0.6972 (6)	0.6680 (6)	0.0664 (4)	0.0368 (11)	
C7	0.4176 (6)	0.7809 (6)	−0.0387 (4)	0.0421 (13)	
C6	0.4559 (6)	0.7750 (5)	0.0747 (3)	0.0294 (10)	
N2	0.3612 (5)	0.8179 (4)	0.1353 (3)	0.0323 (9)	
C12	0.2280 (6)	0.8637 (6)	0.0849 (4)	0.0437 (13)	
H8	0.1601	0.8895	0.1249	0.052*	
C11	0.1825 (7)	0.8757 (7)	−0.0271 (4)	0.0565 (16)	
H7	0.0883	0.9110	−0.0592	0.068*	
C10	0.2789 (7)	0.8348 (7)	−0.0871 (4)	0.0524 (15)	
H6	0.2516	0.8431	−0.1607	0.063*	
C5	0.6006 (6)	0.7203 (5)	0.1290 (4)	0.0299 (10)	
C15	0.6463 (5)	1.1743 (5)	0.4079 (4)	0.0284 (10)	
C13	0.3479 (6)	0.5207 (5)	0.3230 (4)	0.0327 (11)	
C8	0.5223 (7)	0.7281 (7)	−0.0976 (4)	0.0534 (15)	
H5	0.4986	0.7328	−0.1718	0.064*	
O6	0.7627 (4)	1.0478 (4)	0.6690 (2)	0.0336 (7)	
C14	0.2753 (7)	0.3534 (6)	0.3168 (5)	0.0506 (14)	
H9A	0.1575	0.3255	0.2737	0.076*	
H9B	0.2959	0.3470	0.3927	0.076*	
H9C	0.3264	0.2814	0.2800	0.076*	
C16	0.7559 (6)	1.3374 (6)	0.4417 (4)	0.0422 (12)	
H10A	0.8097	1.3412	0.3886	0.063*	
H10B	0.8385	1.3612	0.5172	0.063*	
H10C	0.6894	1.4140	0.4409	0.063*	
C9	0.6513 (7)	0.6730 (7)	−0.0494 (4)	0.0539 (15)	
H4	0.7136	0.6367	−0.0914	0.065*	
C18	0.9975 (5)	0.9554 (6)	0.6560 (4)	0.0395 (12)	
H11A	1.0003	0.8457	0.6445	0.059*	
H11B	1.0414	1.0106	0.7365	0.059*	
H11C	1.0636	1.0025	0.6206	0.059*	
C17	0.8220 (5)	0.9669 (5)	0.6038 (4)	0.0277 (10)	

### Atomic displacement parameters (Å^2^)

**Table:**

	*U*^11^	*U*^22^	*U*^33^	*U*^12^	*U*^13^	*U*^23^
Eu1	0.02392 (12)	0.02795 (13)	0.02150 (12)	0.00926 (9)	0.01093 (9)	0.00800 (9)
O3	0.0368 (18)	0.0376 (18)	0.0297 (16)	0.0146 (15)	0.0192 (14)	0.0159 (15)
O4	0.0410 (19)	0.0386 (19)	0.0242 (16)	0.0079 (15)	0.0181 (14)	0.0077 (15)
O2	0.039 (2)	0.038 (2)	0.045 (2)	0.0085 (16)	0.0110 (16)	0.0142 (17)
O1	0.038 (2)	0.0360 (19)	0.0442 (19)	0.0151 (15)	0.0170 (16)	0.0167 (16)
O5	0.0288 (17)	0.0393 (19)	0.0243 (16)	0.0108 (14)	0.0111 (13)	0.0056 (14)
N1	0.030 (2)	0.028 (2)	0.0260 (19)	0.0091 (16)	0.0132 (16)	0.0064 (16)
C1	0.044 (3)	0.038 (3)	0.032 (3)	0.017 (2)	0.019 (2)	0.011 (2)
C2	0.034 (3)	0.047 (3)	0.050 (3)	0.018 (2)	0.018 (2)	0.013 (3)
C3	0.046 (3)	0.047 (3)	0.048 (3)	0.016 (3)	0.031 (3)	0.004 (3)
C4	0.043 (3)	0.037 (3)	0.032 (3)	0.011 (2)	0.021 (2)	0.003 (2)
C7	0.050 (3)	0.048 (3)	0.025 (2)	0.007 (3)	0.014 (2)	0.010 (2)
C6	0.036 (3)	0.027 (2)	0.022 (2)	0.004 (2)	0.011 (2)	0.0032 (19)
N2	0.034 (2)	0.035 (2)	0.027 (2)	0.0104 (18)	0.0096 (17)	0.0088 (18)
C12	0.039 (3)	0.061 (4)	0.031 (3)	0.024 (3)	0.011 (2)	0.012 (3)
C11	0.064 (4)	0.074 (4)	0.033 (3)	0.033 (3)	0.009 (3)	0.021 (3)
C10	0.069 (4)	0.060 (4)	0.027 (3)	0.018 (3)	0.013 (3)	0.016 (3)
C5	0.037 (3)	0.025 (2)	0.028 (2)	0.005 (2)	0.015 (2)	0.005 (2)
C15	0.027 (2)	0.034 (3)	0.031 (2)	0.012 (2)	0.014 (2)	0.015 (2)
C13	0.043 (3)	0.033 (3)	0.029 (2)	0.012 (2)	0.024 (2)	0.006 (2)
C8	0.067 (4)	0.071 (4)	0.029 (3)	0.018 (3)	0.027 (3)	0.015 (3)
O6	0.0333 (18)	0.0416 (19)	0.0257 (16)	0.0171 (15)	0.0109 (14)	0.0052 (15)
C14	0.064 (4)	0.033 (3)	0.057 (3)	0.004 (3)	0.027 (3)	0.016 (3)
C16	0.049 (3)	0.036 (3)	0.046 (3)	0.007 (2)	0.027 (3)	0.009 (2)
C9	0.063 (4)	0.066 (4)	0.037 (3)	0.017 (3)	0.030 (3)	0.008 (3)
C18	0.030 (3)	0.044 (3)	0.041 (3)	0.013 (2)	0.009 (2)	0.009 (2)
C17	0.027 (2)	0.028 (2)	0.034 (3)	0.0086 (19)	0.014 (2)	0.014 (2)

### Geometric parameters (Å, °)

**Table:**

Eu1—O3^i^	2.358 (3)	C7—C10	1.384 (7)
Eu1—O6^i^	2.374 (3)	C7—C6	1.415 (6)
Eu1—O5	2.377 (3)	C7—C8	1.438 (7)
Eu1—O2	2.453 (3)	C6—N2	1.355 (5)
Eu1—O1	2.470 (3)	C6—C5	1.448 (6)
Eu1—O4	2.513 (3)	N2—C12	1.311 (6)
Eu1—O3	2.586 (3)	C12—C11	1.411 (7)
Eu1—N1	2.594 (3)	C12—H8	0.9300
Eu1—N2	2.649 (4)	C11—C10	1.358 (7)
Eu1—C13	2.815 (5)	C11—H7	0.9300
Eu1—C15	2.920 (4)	C10—H6	0.9300
Eu1—Eu1^i^	3.9409 (8)	C15—C16	1.500 (6)
O3—C15	1.276 (5)	C13—C14	1.508 (6)
O3—Eu1^i^	2.358 (3)	C8—C9	1.324 (8)
O4—C15	1.245 (5)	C8—H5	0.9300
O2—C13	1.262 (6)	O6—C17	1.273 (5)
O1—C13	1.262 (5)	O6—Eu1^i^	2.374 (3)
O5—C17	1.256 (5)	C14—H9A	0.9600
N1—C1	1.319 (6)	C14—H9B	0.9600
N1—C5	1.351 (5)	C14—H9C	0.9600
C1—C2	1.402 (6)	C16—H10A	0.9600
C1—H1	0.9300	C16—H10B	0.9600
C2—C3	1.352 (7)	C16—H10C	0.9600
C2—H2	0.9300	C9—H4	0.9300
C3—C4	1.399 (7)	C18—C17	1.492 (6)
C3—H3	0.9300	C18—H11A	0.9600
C4—C5	1.410 (6)	C18—H11B	0.9600
C4—C9	1.437 (7)	C18—H11C	0.9600
			
O3^i^—Eu1—O6^i^	75.03 (10)	C5—N1—Eu1	120.7 (3)
O3^i^—Eu1—O5	76.96 (10)	N1—C1—C2	123.7 (4)
O6^i^—Eu1—O5	137.07 (10)	N1—C1—H1	118.2
O3^i^—Eu1—O2	86.29 (10)	C2—C1—H1	118.2
O6^i^—Eu1—O2	81.08 (11)	C3—C2—C1	118.2 (5)
O5—Eu1—O2	128.67 (11)	C3—C2—H2	120.9
O3^i^—Eu1—O1	77.36 (10)	C1—C2—H2	120.9
O6^i^—Eu1—O1	127.36 (11)	C2—C3—C4	120.5 (4)
O5—Eu1—O1	75.84 (10)	C2—C3—H3	119.8
O2—Eu1—O1	53.10 (11)	C4—C3—H3	119.8
O3^i^—Eu1—O4	125.07 (10)	C3—C4—C5	117.4 (4)
O6^i^—Eu1—O4	90.28 (11)	C3—C4—C9	123.4 (5)
O5—Eu1—O4	79.96 (10)	C5—C4—C9	119.2 (5)
O2—Eu1—O4	144.17 (10)	C10—C7—C6	117.6 (5)
O1—Eu1—O4	141.79 (10)	C10—C7—C8	123.9 (5)
O3^i^—Eu1—O3	74.40 (11)	C6—C7—C8	118.5 (5)
O6^i^—Eu1—O3	72.72 (10)	N2—C6—C7	122.5 (4)
O5—Eu1—O3	68.79 (10)	N2—C6—C5	118.0 (4)
O2—Eu1—O3	150.59 (10)	C7—C6—C5	119.5 (4)
O1—Eu1—O3	138.62 (10)	C12—N2—C6	117.9 (4)
O4—Eu1—O3	50.80 (9)	C12—N2—Eu1	122.8 (3)
O3^i^—Eu1—N1	143.33 (11)	C6—N2—Eu1	118.7 (3)
O6^i^—Eu1—N1	139.90 (10)	N2—C12—C11	123.2 (5)
O5—Eu1—N1	77.84 (10)	N2—C12—H8	118.4
O2—Eu1—N1	89.07 (11)	C11—C12—H8	118.4
O1—Eu1—N1	70.92 (11)	C10—C11—C12	118.8 (5)
O4—Eu1—N1	75.40 (10)	C10—C11—H7	120.6
O3—Eu1—N1	119.64 (10)	C12—C11—H7	120.6
O3^i^—Eu1—N2	149.00 (11)	C11—C10—C7	120.0 (5)
O6^i^—Eu1—N2	77.11 (11)	C11—C10—H6	120.0
O5—Eu1—N2	133.80 (10)	C7—C10—H6	120.0
O2—Eu1—N2	76.19 (11)	N1—C5—C4	122.1 (4)
O1—Eu1—N2	110.07 (11)	N1—C5—C6	118.6 (4)
O4—Eu1—N2	67.99 (10)	C4—C5—C6	119.3 (4)
O3—Eu1—N2	109.76 (10)	O4—C15—O3	120.4 (4)
N1—Eu1—N2	62.79 (11)	O4—C15—C16	121.0 (4)
O3^i^—Eu1—C13	79.18 (11)	O3—C15—C16	118.5 (4)
O6^i^—Eu1—C13	103.79 (13)	O4—C15—Eu1	58.8 (2)
O5—Eu1—C13	102.11 (13)	O3—C15—Eu1	62.3 (2)
O2—Eu1—C13	26.59 (12)	C16—C15—Eu1	172.3 (3)
O1—Eu1—C13	26.61 (12)	O2—C13—O1	121.4 (4)
O4—Eu1—C13	154.90 (11)	O2—C13—C14	119.9 (4)
O3—Eu1—C13	153.35 (11)	O1—C13—C14	118.8 (5)
N1—Eu1—C13	80.57 (12)	O2—C13—Eu1	60.5 (2)
N2—Eu1—C13	94.65 (12)	O1—C13—Eu1	61.3 (2)
O3^i^—Eu1—C15	99.99 (11)	C14—C13—Eu1	173.6 (3)
O6^i^—Eu1—C15	82.87 (11)	C9—C8—C7	122.1 (5)
O5—Eu1—C15	70.72 (11)	C9—C8—H5	119.0
O2—Eu1—C15	160.62 (12)	C7—C8—H5	119.0
O1—Eu1—C15	146.09 (11)	C17—O6—Eu1^i^	136.1 (3)
O4—Eu1—C15	25.08 (10)	C13—C14—H9A	109.5
O3—Eu1—C15	25.89 (10)	C13—C14—H9B	109.5
N1—Eu1—C15	96.31 (11)	H9A—C14—H9B	109.5
N2—Eu1—C15	89.71 (11)	C13—C14—H9C	109.5
C13—Eu1—C15	172.71 (13)	H9A—C14—H9C	109.5
O3^i^—Eu1—Eu1^i^	39.20 (7)	H9B—C14—H9C	109.5
O6^i^—Eu1—Eu1^i^	69.54 (7)	C15—C16—H10A	109.5
O5—Eu1—Eu1^i^	68.14 (7)	C15—C16—H10B	109.5
O2—Eu1—Eu1^i^	122.20 (8)	H10A—C16—H10B	109.5
O1—Eu1—Eu1^i^	111.19 (7)	C15—C16—H10C	109.5
O4—Eu1—Eu1^i^	85.93 (7)	H10A—C16—H10C	109.5
O3—Eu1—Eu1^i^	35.19 (6)	H10B—C16—H10C	109.5
N1—Eu1—Eu1^i^	143.56 (8)	C8—C9—C4	121.4 (5)
N2—Eu1—Eu1^i^	137.30 (8)	C8—C9—H4	119.3
C13—Eu1—Eu1^i^	118.30 (9)	C4—C9—H4	119.3
C15—Eu1—Eu1^i^	60.89 (9)	C17—C18—H11A	109.5
C15—O3—Eu1^i^	160.5 (3)	C17—C18—H11B	109.5
C15—O3—Eu1	91.8 (3)	H11A—C18—H11B	109.5
Eu1^i^—O3—Eu1	105.60 (10)	C17—C18—H11C	109.5
C15—O4—Eu1	96.1 (2)	H11A—C18—H11C	109.5
C13—O2—Eu1	92.9 (3)	H11B—C18—H11C	109.5
C13—O1—Eu1	92.1 (3)	O5—C17—O6	125.1 (4)
C17—O5—Eu1	139.2 (3)	O5—C17—C18	117.4 (4)
C1—N1—C5	118.2 (4)	O6—C17—C18	117.5 (4)
C1—N1—Eu1	120.9 (3)		

Symmetry codes: (i) −*x*+1, −*y*+2, −*z*+1.

### Hydrogen-bond geometry (Å, °)

**Table:**

*D*—H···*A*	*D*—H	H···*A*	*D*···*A*	*D*—H···*A*
C2—H2···O2^ii^	0.93	2.57	3.287 (6)	135
C12—H8···O6^i^	0.93	2.44	3.078 (6)	126
C16—H10C···O1^iii^	0.96	2.45	3.390 (6)	165

Symmetry codes: (ii) *x*+1, *y*, *z*; (i) −*x*+1, −*y*+2, −*z*+1; (iii) *x*, *y*+1, *z*.
